# Effect of calcium on anxiolytic activity of diazepam and verapamil in rats

**DOI:** 10.4103/0253-7613.71889

**Published:** 2010-12

**Authors:** Sharanabasayyaswamy B. Hiremath, Sohit Anand, L.D. Srinivas, Mohammad Rafiuddin Rashed

**Affiliations:** Department of Pharmacology, J.J.M. Medical College, Davangere, Karnataka, India

**Keywords:** Calcium, calcium channel antagonist, light–dark arena

## Abstract

**Objective:**

To analyze the role of calcium in anxiety and its effect on anxiolytic activity of diazepam and verapamil.

**Materials and Methods:**

Study was conducted using female albino rats in light and dark arena; a nonconflicting animal experimental model for anxiety. Animals were divided into six groups with six animals in each group. Test drugs, calcium gluconate (10 mg/kg), diazepam (1 mg/kg), verapamil (5 mg/kg), calcium + diazepam, and calcium + verapamil were administered intraperitoneally. Percentage of time spent in light arena and number of entries into light arena were the two parameters observed for 5 min after 30 min of drug administration. ANOVA test was used for statistical analysis.

**Results:**

Compared to the control group, diazepam group, and calcium group, only calcium + diazepam group showed considerable increase in mean percentage of time spent in light arena. However, this increase was statistically insignificant. In the case of total number of entries into light arena, animals in calcium + diazepam group showed statistically significant increase in total number of entries into light arena when compared to calcium group and diazepam group.

**Conclusion:**

Results of the study suggest that calcium may enhance the anxiolytic activity of diazepam, but has no effect on anxiolytic activity of verapamil.

## Introduction

Calcium is one of the most important second messengers, and plays a major role in many of the neuropsychiatry disorders; especially mood disorders.[1] Apart from calcium, magnesium is an another divalent cation which also has an important role in psychosomatic disorders.[2,3] Clinical studies on treatment of panic disorders with calcium channel antagonists and preclinical studies on anxiolytic activity of calcium channel antagonists have yielded mixed results.[1,4,5] There are isolated incidences and stray reports of relief of anxiety symptoms after administration of calcium for prevention or treatment of osteoporosis in few patients. Exposure to stress and anxiety are associated with partial magnesium reduction and increased urinary magnesium and calcium excretion.[6] Therefore, the role of calcium in anxiety and anxiolytic efficacy of calcium channel antagonists remains inconclusive and needs further evaluation.

This study was aimed at analyzing the role of calcium in anxiety and its effects on anxiolytic activity of diazepam (a benzodiazepine hypnotic) and verapamil (a calcium channel antagonist) in the animal model of anxiety.

## Materials and Methods

The study was conducted in female albino rats weighing 150–250 g reared in an animal house maintained at optimal temperature and 12 h light–dark cycle with free access to food and water. Animals were divided into six groups of six animals each. The groups comprised of the control group, diazepam group, calcium gluconate group, verapamil group, calcium + diazepam group, and calcium + verapamil group. All the drugs were prepared either by dissolving or suspending in distilled water and administered intraperitoneally. Test drugs, calcium gluconate (10 mg/kg b.w.), diazepam (1 mg/kg body weight), verapamil (5 mg/kg b.w.), were freshly prepared and administered 30 min prior to the test. In animals belonging to calcium + diazepam and calcium + verapamil group, calcium gluconate was administered initially, followed by diazepam or verapamil after 30 min. Control animals received 1 ml/kg b.w. of sterile distilled water intraperitoneally. Doses of diazepam and verapamil used in this study were the doses employed in the previous studies.[1,2] Dose of calcium gluconate selected here was the dose used in one of the preclinical studies to test the analgesic activity of the calcium.[7]

Light and dark arena, a non-conflicting animal experimental model for anxiety was used for testing anxiolytic activity. The apparatus consisted of an open top wooden box with two distinct chambers of specific dimensions connected by a small door measuring 7.5 × 5 cm^2^ at the floor level in the center of partition wall. The dark chamber measuring 20 × 30 × 35 cm^3^ was painted black and illuminated with dimmed red light. The light chamber measuring 30 × 30 × 35 cm^3^ was painted white and illuminated with 100 W bright light located 17 cm above the box. Each rat was placed in the center of the light arena of the apparatus and was allowed to explore it for 5 min. Percentage of time spent in light arena and number of entries into light arena were the two parameters observed for 5 min after 30 min of drug administration. Analysis of variance (ANOVA) test followed by Bonferroni’s *post hoc* test was used for statistical analysis.

## Results

Animals in control group appeared to have shown more than usual exploratory behavior. There were no statistically significant changes in percentage of time spent in light arena, after different treatments as compared to control value. Similarly, treatment with calcium + verapamil did not significantly alter the percentage of time spent in light arena as compared to either calcium or verapamil treatment groups alone [Table 1]. Combined treatment consisting of calcium + diazepam produced considerable increase in mean percentage of time spent in light arena; however, this increase was statistically insignificant [Table 1].

**Table 1:** Effects of different treatment groups on percentage of time spent and number of entries into light arena in light/dark box arena

*Treatment (mg/kg, i.p.)*	*n*	*Percentage time spent in light arena (Mean ± SEM)*	*Number of entries into light arena (Mean ± SEM)*
Control (distilled water) (1 ml/kg, i.p.)	6	9.97 ± 3.83	1.330 ± 0.61
Diazepam (1)	6	13.20 ± 4.18	1.170 ± 0.60
Verapamil (5)	6	8.68 ± 2.89	0.667 ± 0.42
Calcium (10)	6	11.30 ± 3.84	0.833 ± 0.40
Calcium + diazepam (10 + 1)	6	23.40 ± 4.70	3.500 ± 0.67*
Calcium + verapamil (10+5)	6	13.60 ± 3.97	1.830 ± 0.47

* *P* ≤ 0.05 vs. diazepam, calcium (ANOVA followed by Bonferroni’s test).

As in the first parameter, there was a similar trend observed in total number of entries into light arena. There was no statistically significant change in number of entries after different treatments as compared to control group. The combined treatment with calcium + verapamil did not significantly alter the total number of entries into light arena as compared to only calcium and verapamil treatment groups. When compared to treatment with calcium or diazepam alone, combined treatment with calcium + diazepam produced statistically significant increase in the total number of entries into light arena.

## Discussion

Few findings of this study results are controversial and unexpected. There is a lack of statistically significant difference between control group and diazepam group. This is probably because animals in control group might have exhibited more than usual exploratory behavior. In our study, the calcium, channel antagonist, and verapamil did not show anxiolytic activity. Compared to control group, none of the other groups showed statistically significant difference in mean percentage of time spent in light arena. Compared to calcium or diazepam alone, mean percentage of time spent in light arena after administration of both calcium and diazepam was considerably high. Although there was a small increase in mean percentage of time spent in light arena in calcium + verapamil group, this increase appears to convey nothing significant. The results suggest that calcium itself has no anxiolytic activity. It probably enhances the anxiolytic activity of benzodiazepines and has no effect on anxiolytic activity if any, of calcium channel antagonists if so ever they have such effects. But basically, calcium is considered as an exitatory ion and benzodiazepines mediate their anxiolytic activity through GABA receptors.[8] In addition, becauase calcium channel antagonists have been shown to have anxiolytic activity,[4] the biological plausibility of our study results needs to be answered

Elucidation of exact site and mechanism behind the enhanced anxiolytic activity of benzodiazepines by calcium needs further studies. Site of action or interaction of calcium with benzodiazepines is less likely to be peripheral, i.e., on skeletal muscles as there was no decrease, rather there was an increase in number of entries into light arena, thereby ruling out the possibility of effect on tone of the skeletal muscles and skeletal muscle relaxation. Considering the role of calcium in neuropsychiatric disorders in humans and importance of voltage-dependent calcium channels (VDCCs) in physiological functions of the nervous system, it is imperative to speculate that the central nervous system may be the site of interaction between calcium and benzodiazapines.[1,9] Further, the finding that L-type calcium channels found in amygdala play an important role in cued fear conditioning suggests that calcium is an important mediator of anxiety.[10] The possibility of interaction between benzodiazepines and calcium is supported by the fact that benzodiazepines mediate some of their pharmacological actions in nervous system through calcium channels.[11,12] The finding that nifedipine blocks the hypnotic effect of flurazepam (a benzodiazepine) is suggestive of benzodiazepines mediating their anxiolytic activity through calcium channels.[11] Unlike at the other sites where nifedipine blocks the entry of calcium by blocking calcium channels, it has paradoxical effect of facilitation of ^45^Ca (radioisotope calcium) uptake by rat hippocampal synaptosomes; supposed to be operated by tetrodotoxin-sensitive Na^+^channels.[13] Thus, the overall evidences suggest that calcium may enhance the anxiolytic activity of benzodiazepines. In our view, although the involvement of GABA receptors and direct actions of the calcium on modulation of neurotransmitter release or nerve excitability is less likely to be responsible for this enhanced effect; it needs further studies to confirm the above suggestion.

Uncertainty regarding the validity of animal models of anxiety and lack of relevance of the study findings to humans are the limitations of our study as are those of any preclinical study; especially those related to neuropsychiatry. Further studies employing different experimental models and different doses of calcium are needed to evaluate the role of calcium in anxiety and its effect on anxiolytic activity of benzodiazepines. This study may help in future for better understanding of anxiolytic activity of benzodiazepines and thus development of newer drugs and newer modalities of treatment.
